# A novel activity on thymocytes cells exerted by the rattlesnake (*Crotalus durissus cumanensis*) venom

**DOI:** 10.7705/biomedica.5599

**Published:** 2021-09-22

**Authors:** María M. Pulido-Méndez, Elvia Azuaje, Alexis Rodríguez-Acosta

**Affiliations:** 1 Laboratorio de Inmunología, Instituto de Medicina Experimental, Universidad Central de Venezuela, Caracas, República Bolivariana de Venezuela Universidad Central de Venezuela Instituto de Medicina Experimental Universidad Central de Venezuela Caracas Venezuela; 2 Laboratorio de Inmunoquímica y Ultraestructura, Instituto Anatómico, Universidad Central de Venezuela, Caracas, República Bolivariana de Venezuela Universidad Central de Venezuela Instituto Anatómico Universidad Central de Venezuela Caracas Venezuela

**Keywords:** Crotalus, crotalid venoms, thymocytes, monocytes, macrophages, venom, Crotalus, venenos de crotálidos, timocitos, monocitos, macrófagos

## Abstract

**Introduction:**

The thymus is active mainly during the neonatal and pre-adolescent periods.

**Objective:**

To test naïve thymocytes proliferation and monocytes stimulation.

**Materials and methods:**

We collected fresh thymus tissue from neonate mice after surgery. Suspension cells were coated onto Ficoll-Hypaque support. The obtained cells (thymocytes) were cultured measuring the proliferation of naïve T cells stimulated by *Crotalus durissus cumanensis (Cdc)* venom at sub-lethal doses (20 ng). Then, we supplemented the wells with AlamarBlue™ and incubated them for 5 h to test their proliferation. Mononuclear cells from mice peripheral blood were collected and layered onto the support of the Ficoll-Hypaque solution. We added the thymocytes actively dividing (25 x 10^5^ cells) from cultures stimulated with *Cdc* venom at 20 ng/well to cultured monocytes freshly obtained from the Ficoll-Hypaque separation. Both cell populations were incubated for 36 h until monocytes matured to macrophages.

**Results:**

The naïve thymocytes rapidly proliferated after stimulation with the *Cdc* venom (NT*Cdc)* and these successively induced the maturation and function of monocytes progenitor cells to mature macrophages, which ingested Chinese ink.

**Conclusions:**

The naïve thymocytes proliferated by stimulation with the *Cdc* venom and subsequently the NT*/Cdc* induced the rapid maturation and function of monocytes progenitor cells becoming mature macrophages with their phenotypic characteristics.

## Resumen

**Introducción.:**

El timo es activo principalmente durante los períodos neonatal y preadolescente.

**Objetivo.:**

Probar la proliferación de los timocitos tempranos y la estimulación de monocitos que producen.

**Materiales y métodos.:**

Se recogió tejido de timo fresco después de la cirugía de ratones recién nacidos. La suspensión de células se colocó sobre un soporte de Ficoll-Hypaque. Las células obtenidas (timocitos) se cultivaron y se midió la proliferación de células T vírgenes estimuladas por el veneno de *Crotalus durissus cumanensis (Cdc)* en dosis subletales (20 ng). A continuación, se agregó AlamarBlue™ a los pocillos y se incubaron durante 5 horas para evaluar la proliferación. Se recogieron células mononucleares de sangre periférica de ratones y se colocaron sobre un soporte de solución de Ficoll-Hypaque. Los timocitos que se dividieron activamente (25 x 10^5^ células) a partir de los cultivos estimulados con veneno de *Cdc* (20 ng/pocillo) y se agregaron a los cultivos de monocitos recién obtenidos de la separación en la solución de Ficoll-Hypaque. Ambas poblaciones celulares se incubaron durante 36 horas hasta que los monocitos maduraron a macrófagos.

**Resultados:**

Los timocitos tempranos experimentaron una rápida proliferación estimulada por el veneno de *Cdc* (NT*Cdc)* y, posteriormente, indujeron la maduración y la función de las células progenitoras de monocitos, los cuales maduraron a macrófagos, que se tiñeron con tinta china.

**Conclusiones.:**

Los timocitos tempranos proliferaron con la estimulación del veneno de *Cdc* y, posteriormente, el NT/Cdc indujo la maduración rápida y la función de las células progenitoras de monocitos, transformándose en macrófagos con sus características fenotípicas.

The thymus gland is located in the mediastinum, at the back of the sternum bone. It is structured by two lobes, which are divided into a central medulla and a peripheral cortex. The thymus is active mainly during the neonatal and pre-adolescent periods ^1^. During adolescence, the organ practically ceases to exist until it weighs around 5 g and is substituted by fat tissue. Subsequently, naïve T cells output is reduced harshly declining the immune response to freshly met antigens, which renders thymus involution a major cause of age-related deterioration in the immune system activity. The thymus is a crucial organ for the maintenance of the immune system. It controls and regularizes the total immune system and its organization in mammals. Together with bone marrow, the thymus is the primary donor of cells for the lymphatic system ^2^. In its interior, progenitor cells are formed maturing and differentiating into mature T cells ^3^. A naïve T cell is a pre-T cell that has been differentiated into bone marrow and effectively suffered the positive and negative routes of central selection in the thymus, among which we find the naïve forms of cytotoxic T cells (CD8+) and helper T cells (CD4+) ^4^.

The possibility of enhancing the thymus function may prove important in a wide variety of immunological diseases and, therefore, the development of new approaches to regenerate the matured thymus is very valuable ^1^^,^^5^. In the past, several authors ^6^^)^ demonstrated that thymus epithelial cells released humoral factors granting T cell precursors (thymus-dependent lymphocytes) the ability to differentiate and maturate into rather mature T cells. They showed that acetylcholine (Ach) boosted the proliferation of thymus epithelial cells, but this finding was totally surpassed by the pre-treatment of alpha-bungarotoxin (alpha-BTx), one of the components of the Taiwanese krait snake *(Bungarus multicinctus)* venom ^7^.

In this context, we wanted to explore the autochthonous *Crotalus durissus cumanensis (Cdc)* snake venom to see if it stimulated α-bungarotoxin-like activities. We proposed that dividing Cdc-venom-stimulated naïve thymocytes (NTCdc) would regulate the maturation and function of monocytes progenitor cells increasing their differentiation and then rapidly developing into mature macrophages. Our aim, then, was to test the *Cdc* venom capability to stimulate the division of naïve thymus cells, which, in their turn, would be able to induce T-lymphocytes and monocytes maturation.

## Materials and methods

### Reagents

Ficoll 400 was purchased from Pharmacia Fine Chemicals, Uppsala, Sweden, and Hypaque (50% w/v) from Winthrop-Breon Laboratories, MO, USA; minimum essential media (MEM) and fetal calf serum, from GIBCO (Thermo-Fisher Scientific, USA), and alamarBlue™ (resazurin:7-hydroxy-3H-phenoxazine-3-one-10-oxide sodium salt), from Sigma-Aldrich (St. Louis, MO, USA).

### Mice

We obtained the congenic C57BL/6c-strain female mice (6 weeks) from the laboratory animal center at *Instituto Venezolano de Investigaciones Científicas* (IVIC). The animals were sacrificed by intraperitoneal injection of Pentothal™ following norms and international procedures for the use of laboratory animals ^8^.

### Venom

The *Cdc* venom was obtained from snake specimens kept at the immunochemistry and ultrastructural laboratory at *Instituto Anatómico, Universidad Central de Venezuela,* Caracas, originated at Santa Teresa del Tuy, Miranda State, República Bolivariana de Venezuela. *Cdc* expected dilutions in 0.5 mg/ml concentrations were preserved in aliquots at - 30° C until use.

### Thymus tissue isolation

Fresh thymus tissue was collected from neonate mice after surgery and the cells were isolated immediately. After eliminating the blood vessels and the capsule, the thymus was washed thoroughly with MEM but no fetal calf serum (FCS). The organ was then cut into 1 mm^3^ fragments and then broken up in cold in Petri dishes on slush ice to free the cells; all cell suspensions were moved to a sterile 50-ml centrifuge tube by passing it through a wire mesh filter to remove the remains of tissue and undisintegrated cells. Next, we added 5 ml of MEM (without FCS) to recover the top number of cells and relocate them in the 50-ml tube. The volume was taken to 30 ml with MEM solution to continue the washing by centrifugation (1,800g for 10 min) at 10°C and get the cell pellet with the thymocytes.

### Thymocytes isolation

The suspension of cells previously isolated was diluted with an equal volume of 0.9% (w/v) NaCl, then coated onto Ficoll-Hypaque support (1.077 g/ml) and centrifuged at room temperature for 30 min at 400*g.* The cells were collected from the interface and washed twice with 20 mM phosphate buffer saline (PBS), pH 7.4.

We used a hemocytometer for the counting of the cells obtained while their viability was verified by trypan blue dye exclusion (Sigma, Mo, USA). The cell counting tubes contained 20 μl of cell suspension diluted in 30 μl of MEM (without FCS) solution to which 50 μl of exclusion dye were added (dilution factor = 5). After 5 to 15 min, we conducted the counting and checked cell viability assumed to be acceptable above 95%. Subsequently, we added 200 Ul (6 x 10^6^ cells) of cell suspension in MEM with 10% FCS complemented with antibiotics (streptomycin 100 μg/ml; penicillin 100 lU/ml) per well and incubated at 37^0^C in a 5% CO_2_ humidified incubator.

### Naïve-thymocytes stimulated with Cdc venom (NTCdc)

We cultured thymocytes (1 x 10^6^ cells/well) and measure the proliferation of naïve T cells stimulated with *Cdc* venom at sub-lethal doses (20 ng). The counts mean was estimated and plotted against culture time producing a growth curve using the AlamarBlue™ (resazurin) proliferation assay.

### AlamarBlue™ (resazurin) proliferation assay

For this assay, we poured 1 x 10^4^ thymocytes per well into 96-microtitre plates to determine cell proliferation with 200 μl of MEM. After adaptation, cells were treated with *Cdc* venom (20 ng/well). After 36 h of exposure, the medium was extracted and the cells were washed with 300 μl of PBS (-Mg/-Ca). Then, 200 μl of fresh MEM having 2 mM AlamarBlue™ were supplemented to the wells and then wrapped in aluminum foil and incubated for 5 h at 37^0^C, 5% CO_2_, and 95% humidity. We included cells without venom as controls. Afterwards, 160 μl aliquots of each sample and controls were relocated into white fluorescence measurement plates (Falcon™, Corning, NY, USA). The amount of reduced AlamarBlue™ (fluorescence signals) was proportional to the proliferative activity of the cells and expressed as a percentage. To calculate the reduction percentage, we read cultures' absorbance at Xex = 570 and Xem = 600 nm wavelengths where the absorption peaks of the reduced and oxidized forms occurred. After subtracting the values of the blanks from the medium, we calculated the average of the triplicates, the oxide-reduction correction factor (Ro), and the percentage of reduced AlamarBlue™ in each experimental condition.

### Monocytes isolation

We diluted mice peripheral blood collected by using sodium citrate as anti-coagulant with an equal volume of 0.9% (w/v) NaCl layered onto a Ficoll-Hypaque support (1.077 g/ml) and centrifuged at room temperature for 30 min at 400g. Monocytes were purified from mononuclear cells, which were collected from 25 ml of mice peripheral blood (up to 12 x 10^7^ cells) by means of sodium citrate (used as anti-coagulant) diluted with an equal volume of 0.9% (w/v) NaC1, layered onto Ficoll-Hypaque support (1.077 g/ml). We obtained a continuous density gradient by centrifugation where cells were settled into two close-fitting bands, one at the top of the tube and the other at the bottom, separated by a distance of 5 cm. Thus, the collection of the different groups of cells without cross-contamination was easy. The top band contained most of the monocytes while the lymphocytes and some monocytes were present in the bottom band. The mononuclear cells were then collected from the interface and washed twice with 20 mM PBS, pH 7.4. The cells were resuspended in a small volume of PBS. Final cell preparations enclosed regularly monocytes containing an insignificant number of granulocytes namely eosinophils or basophils cells as the main contaminating cells. The monocytes were identified by nonspecific esterase (NSE) staining ^9^. Finally, cells with constant viability > 99% were coated in tissue culture plates (Nunclon® Delta, 24 wells, Denmark) and 10^6^ cells per well in 1 ml MEM complemented with antibiotics (streptomycin 100 μg/rnL; penicillin 100 lU/ml) and 10% FCS and then incubated at 37^0^C in a 5% CO_2_ humidified incubator.

### Thymocytes stimulated with Cdc venom and monocytes experimental culture

Thymocytes actively dividing (25x10^5^ cells) from cultures stimulated with *Cdc* venom (20 ng/well) were added to cultured monocytes freshly obtained from Ficoll-Hypaque separation (1x10^5^ cells/ 200 μl per well) in a plate. Both cell populations were incubated with MEM 10% FCS at 37^0^C and 5% CO_2_ for 36 h until monocytes matured to macrophages.

### Monocyte/macrophage function assay for uptake of Chinese ink

Monocyte/macrophage cultures stimulated with NT*Cdc* and medium with Chinese ink (10 μl/ml) were included on the culture plate, which was then incubated for another 6 h. The cells were observed under the light microscope and then the cells cultured were tested.

### Ethical statement

Experienced staff prepared all the experimental assays involving the use of live animals, which were authorized by the Institute of Anatomy Ethical Committee at *Universidad Central de Venezuela* on March 17, 2019 (minutes 17-03-19). The assays followed the Guide for the Care and Use of Laboratory Animals published by the U.S. National Institutes of Health (NIH Publication No. 85-23, revised 1996) ^8^.

## Results

### The Cdc venom effects on the proliferation of thymus naïve cells

**Figure 1:**
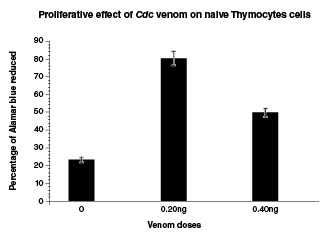
Naïve thymocytes were cultured at 1 x 106 cells with and without *Crotalus durissus cumanensis* venom using 0.20 and 0.40 ng doses. *Crotalus durissus cumanensis* venom (0.20 ng and 0.40 ng)** caused a significant increase in the proportion of naïve t-cells when compared to the control group (0). **p<0.01

Naïve thymus cells exposed for 1 h to sub-lethal doses (20 ng) of *Cdc* venom generated a significant cellular increase of 248.67% compared to the control. This increase using a 40 ng dose decreased to 112.8% compared to the control (figure 1 and table 1); nevertheless, a proliferative effect at 40 ng dose was still observed compared to the control, which suggests that when the dose of *Cdc* venom is increased, the proliferative effect in thymocytes tends to decrease.

**Table 1:** *Crotalus durissus cumanensis* venom effect on thymus cells proliferation

Venom (ng/well)	*C. d. cumanensis*
	% of AlamarBlue™ reduced ± SD
0	23.3 ± 1.6
0.2	80.3 ± 2.4
0.4	49.6 ± 2.7

SD: Standard deviation

**Figure 2:**
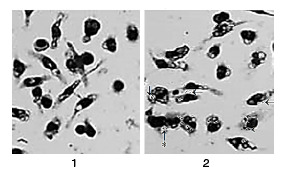
Macrophage cultures. **(1)** Normal control cells. (2) Macrophage with Chinese ink phagocytized (arrows); multinucleated (giant) cell with Chinese ink phagocytized (arrows + asterik). 100X

A substantial proportion of the monocytes cells stimulated by *Cdc* venom (NTCdc) promoted the differentiation to mature macrophages. When cultured in medium solo (normal controls) for 36 h, <18% of monocytes stayed alive, and of those <20% differentiated into macrophages (figure 2). Survival was significantly improved by the addition of naïve thymus cells stimulated by *Cdc* venom (p<0.01) (figure 1 and table 1). The activity of Cdc-venom stimulated naïve thymus cells induced monocytes to differentiate into macrophages characterized by initial morphological and functional changes towards stellate cells adhered to the plastic of the culture plate. Some cells fused and transformed into multinucleated cells (giant cells) with a high capacity to phagocyte particles of Chinese ink (figure 2). Roughly 95% of cell cultures were identified morphologically as monocytes by Wright-Giemsa staining. These cells were also specially stained with markers for nonspecific esterases, markers which were engaged in the monocytes and then differentiated into macrophages subsequently utilized in fresh and mature monocytes for the recognition of monocyte-macrophage cell populations. Morphologically, monocytes began to mature into macrophages soon after they were placed in culture with NT*Cdc.* The analysis of monocytes sections in the plates showed that cell suspensions exhibited some big significant central nuclei bordered by a fine layer of cytoplasm. When the NTCdc cells were added to the cultures, they changed rapidly in the culture (12 h) while nuclei adopted no central location in the cell and cytoplasm augmented in dimensions. Multiple cytoplasm extensions appeared on cell surfaces and subcellular structures such as lysosomes, mitochondria, smooth endoplasmic reticulum, and Golgi apparatus became conspicuous. At 36 h, the macrophages had increased ~6-fold and several phagocytic vesicles were noticed; also, many of these cells had fused to become multinucleated (giant) cells.

### Monocyte/macrophage function assay for uptake of Chinese ink

We carried out the Chinese ink uptake test on day 2 of culture. Most of the cells were positive for Chinese ink uptake demonstrating that they preserved their monocyte/macrophage function during the course of the *in vitro* culture ^9^ (figure 2).

## Discussion

The thymus gland is a supplier of immunocompetent cells mainly during the neonatal period ^10^^,^^11^. Naïve thymocytes change their character and develop the ability to transform in T-lymphocytes. T cells migrate to the periphery and enter the naïve T cell compartment after being selected as positive and negative in the thymus. In the circulatory system, naïve T cells uninterruptedly recirculate between secondary lymphoid organs and plasma via the lymphatic system. When these cells come across and interact with related antigens in the circulatory system, naïve T cells multiply and differentiate into dissimilar types of effector and memory T cells. The naïve T cell section must preserve a large number of cells with unique T cell receptors (TCRs) in a restricted location ^4^. It is in this space that the *Cdc* venom presumably acts in its mitotic stimulation effect.

Previous histopathological studies of mice thymus have exhibited a patent deficiency of lymphocytes in lymphoid organs ^12^ developing a defective immune reactivity. Therefore, the thymus is in charge of the maturation and selection of immunologically competent small lymphocytes ^13^. These thymus cells are involved in the general immune responses ^10^. It is known that small lymphocytes are the cells responsible for protecting the animal organism against extraneous antigens circulating in the circulatory system ^14^. Contact with the *Cdc* venom stimulated a strong NT*Cdc* mitotic activity resulting in an increased cell division in the *in vitro* cultures. It has been demonstrated *in vivo* that the contact with some antigens causes an increase in lymphopoiesis in the peripheral lymphoid organs ^11^^,^^15^ resulting in the mitosis and unexpected reproduction of naïve thymocytes, which later were able to stimulate monocytes maturation into macrophages.

The proportion of macrophages can influence the host's response to tumors, infections, and immune diseases ^16^^,^^17^. In this sense, we describe here the NT/Cdc-mediated maturation of mice monocytes into mature and activated macrophages. The resulting cells were characterized phenotypically by their morphological changes ranging from monocytes rounded cells to stellate macrophage cells with multiple extensions and the functional capacity to mediate augmented endocytosis and phagocytizing particles of Chinese ink. We compared the phenotype and function of macrophages generated by the NT*Cdc* action and found that the populations of normal monocytes and activated macrophages had dissimilar morphologies and generated macrophages with greater endocytic activity.

It is known that the Chinese ink uptake assay is used to estimate the phagocytic function of leukocytes/monocytes ^18^. But as it does not only express the phagocytic function of cells, it may similarly be used to validate the presence of "true macrophages" exhibiting monocyte/macrophage characteristics. Our results showed that the cells were positive for Chinese ink uptake and we observed that the stimulated macrophages kept their monocyte/ macrophage performance throughout the time of the *in vitro* culture ^18^.

We think that NT*Cdc*s have played an important role in the general process of monocyte differentiating into macrophages. The similarity between monocytes stimulated by T-cell immune responses and NTCdc-stimulated macrophages suggests that there must be various regulatory pathways mediating the differentiation of monocytes into macrophages. Some of these pathways have been previously identified in studies of murine monocytes cell lines ^19^^-^^22^.

Thymus involution is the main causal factor of the reduction of the immune response with age since aging produces a gradual decrease in the structural integrity of the thymus ^1^ finally taking to a stable deterioration of undifferentiated T-cell production and an incomplete range of T-cell receptor (TCR) cells. Many of these cells turn into immune memory cells producing an augmented risk of severe immune deficiency due to the lack of naïve T-cells.

Thymocyte cell transplant could represent an applicable way to overhaul the immune system and re-establish its performing actions. However, the central impediment is an absence of supplies for these specific cells ^1^. Thymus deprivation results in serious diseases due to the absence of a suitably operating immune system. Cells resulting from adult thymus contributors do not have similar results, as the gland expires with age. We propose, therefore, that the purification of the *Cdc* venom fraction (s) may have the potential to stimulate the small population of naïve thymocytes left in the adult thymus and induce their proliferation, which, in its turn, would induce the rescue of undifferentiated T-cells the production leading to a normalization of the TCR cells repertoire. The use of this method would be promising to aid newborn infants lacking the thymus, as well as aged individuals.

In conclusion, we propose here that naïve thymocytes rapidly proliferate when stimulated with *Cdc* venom and, subsequently, the NTCdc regulates the rapid maturation and function of monocytes progenitor cells.
